# Erythropoiesis: insights into pathophysiology and treatments in 2017

**DOI:** 10.1186/s10020-018-0011-z

**Published:** 2018-03-23

**Authors:** Andrea Zivot, Jeffrey M. Lipton, Anupama Narla, Lionel Blanc

**Affiliations:** 1 Laboratory of Developmental Erythropoiesis, Center for Autoimmune, Musculoskeletal, and Hematopoietic Diseases, The Feinstein Institute for Medical Research, 350 Community Drive, Manhasset, NY 11030 USA; 2 Division of Pediatrics Hematology/Oncology and Stem Cell Transplantation, Cohen Children’s Medical Center, New Hyde Park, NY 11040 USA; 3 Stanford University School of Medicine, Stanford, CA USA; 4 Department of Molecular Medicine and Pediatrics, Donald and Barbara Zucker School of Medicine at Hofstra Northwell, Hempstead, NY 11549 USA

**Keywords:** Erythropoiesis, Therapy, Red cell disorders

## Abstract

Erythropoiesis is a tightly-regulated and complex process originating in the bone marrow from a multipotent stem cell and terminating in a mature, enucleated erythrocyte.

Altered red cell production can result from the direct impairment of medullary erythropoiesis, as seen in the thalassemia syndromes, inherited bone marrow failure as well as in the anemia of chronic disease. Alternatively, in disorders such as sickle cell disease (SCD) as well as enzymopathies and membrane defects, medullary erythropoiesis is not, or only minimally, directly impaired. Despite these differences in pathophysiology, therapies have traditionally been non-specific, limited to symptomatic control of anemia via packed red blood cell (pRBC) transfusion, resulting in iron overload and the eventual need for iron chelation or splenectomy to reduce defective red cell destruction. Likewise, in polycythemia vera overproduction of red cells has historically been dealt with by non-specific myelosuppression or phlebotomy. With a deeper understanding of the molecular mechanisms underlying disease pathophysiology, new therapeutic targets have been identified including induction of fetal hemoglobin, interference with aberrant signaling pathways and gene therapy for definitive cure. This review, utilizing some representative disorders of erythropoiesis, will highlight novel therapeutic modalities currently in development for treatment of red cell disorders.

## Background

In this review, we hope to provide the reader with an overview of our current understanding of human erythropoiesis, along with a classification of representative disorders leading to a decrease or increase in red cell production with the clinical strategies being used currently or in the near future for management of these patients. The discussion of steady state erythropoiesis provides the framework for understanding these representative disorders and allows the reader to contextualize other diseases, not described herein, within this framework. For a deeper understanding of the molecular bases governing erythropoiesis, the reader is referred to these outstanding reviews published within the last 5 years (Katsumura and Bresnick 2017; An et al. 2015; Palis 2014; Kalfa and Zheng 2014; Crispino and Weiss 2014; Keerthivasan et al. 2011).

## Erythropoiesis at steady state

Every second, the human body generates 2 million red blood cells, through the process of erythropoiesis. Human erythropoiesis is a complex, multi-step process, from the multipotent hematopoietic stem cell (HSC) to the mature erythrocyte (Orkin 2000). The first steps of erythroid differentiation involve an engagement phase, in which HSCs differentiate into more committed erythroid progenitors, from a common myeloid progenitor the megakaryocytic-erythroid progenitor and finally the burst-forming unit- erythroid (BFU-E). BFU-Es are the first progenitor cells committed solely to the erythroid lineage (Gregory and Eaves 1977). These BFU-Es further differentiate into the colony forming unit-erythroid (CFU-E), following which, terminal differentiation occurs.

The second phase of erythroid maturation involves the differentiation of the nucleated precursors from proerythroblasts to basophilic, polychromatophilic and orthochromatic erythroblasts. This phase is characterized by the gradual accumulation of hemoglobin, progressive decrease in cell size and nuclear condensation ultimately resulting in enucleation (Granick and Levere 1964).

The final phase of erythroid development involves the maturation of the reticulocyte into erythrocytes. It is during this stage that the erythrocyte acquires its biconcave shape through extensive membrane remodeling and will circulate in the blood stream until it is removed by the macrophages within the reticuloendothelial system (Gifford et al. 2006).

Terminal erythroid differentiation occurs in anatomic niches known as erythroblastic islands. Erythroblastic islands are unique to mammalian erythropoiesis and consist of a central macrophage surrounded by up to 30 erythroid cells at varying degrees of red cell maturation (Lee et al. 1988). The cells range from CFU-Es to enucleating erythroblasts and are the site of hemoglobin synthesis by terminally differentiating erythroblasts (Bessis 1958; Bessis and Breton-Gorius 1962). The central macrophage functions to anchor erythroblasts within the island and provide the cellular interactions necessary to drive erythroid differentiation and proliferation. Furthermore, the central macrophage has also been shown to phagocytose the extruded nucleus from terminally differentiating erythroblasts (Seki and Shirasawa 1965; Skutelsky and Danon 1972; Bessis et al. 1978) and direct the transfer of iron to erythroid progenitors for heme synthesis (Bessis and Breton-Gorius 1962; Leimberg et al. 2008).

Macrophages within erythroblastic islands also help regulate the rate of erythropoiesis via positive and negative feedback mechanisms. Macrophages secrete cytokines such as insulin-like growth factor-1 that promote erythroid proliferation and maturation (Kurtz et al. 1985; Sawada et al. 1989). Other functions for the central macrophage are still being investigated.

**Fig. 1:**
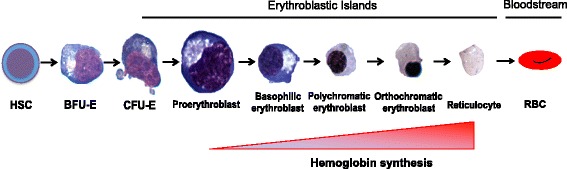
Overview of erythropoiesis, from the hematopoietic stem cell (HSC) to the red blood cell (RBC). Erythropoiesis takes place in the bone marrow, and erythroblastic islands are niches for erythropoiesis from the CFU-E to the reticulocyte state. Then the reticulocyte reaches the blood stream where it achieves its maturation by losing its internal organelles, remodeling its plasma membrane and finally becomes a RBC

At baseline, erythropoiesis occurs at a steady, but low basal rate with approximately 1% of circulating erythrocytes cleared and replaced by new cells daily (Dzierzak and Philipsen 2013). RBCs remain in circulation for approximately 120 days during which time they are continuously surveyed by resident macrophages within the liver and spleen (Crosby 1959). Macrophages within the spleen can detect and remove unwanted or damaged RBCs as well as aged RBCs at the end of their life span (Crosby 1957). Figure 1 provides an overview of human erythropoiesis.

### Regulation of erythropoiesis

A detailed description of the regulation of human erythropoiesis is beyond the scope of the current review. However, several key regulators will be briefly covered as they are involved in the erythropoietic disorders discussed below.

Erythropoietin (EPO) is a humoral cytokine synthesized primarily in the kidney and secreted into the blood stream where it targets erythroid progenitor cells in the bone marrow (Broxmeyer 2013). The primary function of EPO is to regulate oxygen delivery to peripheral tissues and is facilitated by the hypoxic induction of EPO gene transcription. Multiple transcription factors are involved in this process including hypoxia inducible factor, regulated by the relative level of hypoxia, and GATA binding proteins (Bunn 2013). Among the GATA proteins, GATA-1, GATA-2 & GATA-3 have been shown to negatively regulate the expression of EPO mRNA via binding in the EPO promoter region (Imagawa et al. 1997). As such, the rate of EPO gene transcription is regulated by the local oxygen environment. In addition to regulating the expression of EPO mRNA, specifically GATA-1 and GATA-2 play crucial roles in the regulation of lineage-restricted gene expression during erythroid differentiation. GATA-1 is necessary for the survival and terminal differentiation of erythroid progenitors, whereas GATA-2 regulates the maintenance and proliferation of hematopoietic stem and progenitor cells. It is the relative proportion of GATA-1 and GATA-2 expression, that drives expression of target genes necessary to drive erythroid maturation and ultimate expression of β-globin genes (Moriguchi and Yamamoto 2014).

EPO binds to the erythropoietin receptor (EPO-R) which causes receptor homodimerization. Erythropoiesis regulation by EPO is temporally regulated, and human studies have shown that EPO binds to the EPO-R from the CFU-E stage to the polychromatophilic stage (Wu et al. 1995; Koury and Bondurant 1988). One of the main signaling pathways mediated by the EPO/EPO-R interaction is JAK2 activation (Witthuhn et al. 1993), which subsequently phosphorylates and activates STAT5. The JAK2/STAT5 pathway has been shown to activate genes fundamental for erythroid progenitor survival, proliferation and differentiation (Grebien et al. 2008). Furthermore, STAT5 phosphorylation is essential for acceleration of erythropoiesis during times of hypoxic stress. The JAK2/STAT5 pathway is chronically activated in polycythemia vera (Yan et al. 2012) and β-Thalassemia (Libani et al. 2008) by intrinsic (somatic mutations) and extrinsic mechanisms, respectively. Other downstream activation pathways include mitogen-activated protein kinase (MAPK) and phosphoinositide 3-kinase (PI3K). Similarly, these pathways are involved in the differentiation and proliferation of erythroid progenitors (Zhang et al. 2014).

All differentiating erythroblasts express Fas ligand, however only immature erythroblasts, predominately the proerythroblast and basophilic normoblast are vulnerable to Fas/Fas-ligand cross-linking. Mature erythroblasts at the polychromatic and orthochromatic stage, utilize this susceptibility to Fas-ligand-mediated cytotoxicity to promote continued erythroid differentiation (De Maria et al. 1999). There has been speculation that during periods of anemia, high levels of erythropoietin expression protect immature erythroblasts from Fas-ligand mediated cytotoxicity, thus promoting erythroid survival and an increased rate of erythropoiesis (Liu et al. 2006) via the extrinsic as well as the intrinsic cell death pathways.

## Erythropoietic disorders

Altered red cell production can be caused by direct impairment in medullary erythropoiesis as seen in the thalassemia syndromes, the anemia of chronic illness and polycythemia vera, a myeloproliferative bone marrow disorder with disordered erythropoiesis. Conversely other disorders such as the sickle cell disease (SCD) syndromes are presented as an example of anemia characterized by essentially normal medullary erythropoiesis.

### Pathophysiology of β- Thalassemia

Hemoglobin synthesis is controlled by two multi-gene clusters located on chromosome 16 (α-like globins) and chromosome 11 (β-like globins). The α gene product combines with the β gene product to form Hb A (α2β2), which is the major form of adult hemoglobin. During fetal life, two γ genes, also located on chromosome 11, combine with α- globin genes to form Hb F (α2 γ 2). A gradual switch from Hb F to Hb A begins before birth and is largely completed by 6 months of age.

In β-thalassemia, a mutation in the β-globin gene results in an imbalance between α- and β- globin chains. This results in an accumulation of unstable α-tetramers within erythroid cells leading to premature cell death within the differentiating red cell. This leads to ineffective erythropoiesis with impaired differentiation of maturing erythroblasts at the polychromatic and orthochromatic phase)(Centis et al. 2000), and structural-membrane deformities (Yuan et al. 1994; Aljurf et al. 1996). The resultant anemia stimulates a compensatory increase in erythropoiesis, with increased proliferation of erythroid precursors in the bone marrow leading to medullary expansion, bony deformities, extramedullary hematopoiesis, and hepatosplenomegaly. In addition, the ineffective erythropoiesis causes suppression of hepcidin resulting in increased iron absorption and primary iron overload (Rivella 2009).

Almost 200 mutations in the β-globin gene locus have been identified that can cause β-thalassemia. Given this complexity, it is more useful clinically to classify β-thalassemia as major, intermediate or mild based on the severity of anemia and the degree of anemia and physical findings. Thalassemia major presents as severe transfusion-dependent anemia in patients who are compound heterozygous or homozygous for two β^0^ alleles. Thalassemia intermedia is a genetically heterogeneous condition with varying degrees of anemia in which patients may require intermittent transfusions and/or a splenectomy. Patients are usually homozygous for the mild β^+^ thalassemia allele or compound heterozygous for a mild β^+^ thalassemia allele and a severe β^0^ thalassemia allele. Patients with β-thalassemia minor are asymptomatic and usually only mildly anemic although they are likely to have microcytosis. The clinical phenotype of the β-thalassemias can be further modified by additional factors such as elevated levels of HbF or co-inheritance of α-thalassemia, particularly when 2 α chains are deleted resulting in a reduction in the formation of α tetramers, demonstrating the role of α:β chain imbalance in the pathophysiology of the disorder.

β -thalassemia can also be co-inherited with hemoglobin E, a structural β variant that results in Hemoglobin E/β-thalassemia. This disease phenotype can similarly be classified as major, intermediate or mild depending on severity of anemia at presentation. Mutations in α genes are common in the population affected by E/β-thalassemia which serves to mitigate the severity of the thalassemia. Most patients present with a thalassemia intermedia phenotype however major-like conditions with transfusion-dependence as well as very mild forms also do exist (Vijay et al. 2015).

### Pathophysiology of α-thalassemia

α-thalassemia results from the reduction or absence of α-globin chains leading to an excess of β-chains that form a precipitate within the developing red cell. Unbound β-chains form tetramers, termed HbH (β4) in adults or Hb Bart’s (γ4) in the fetal period causing hemolytic anemia and ineffective erythropoiesis. Individuals affected by α-thalassemia have variable degrees of anemia, microcytosis and percentage of HbA_2_ depending on the number of affected non-functional alpha globin genes (1–4 α-globin genes) and the relative proportion of functional α chain synthesis (Kan et al. 1968; Kan and Nathan 1970). α-thalassemia silent-carrier state occurs with a single α-globin gene defect and patients are clinically asymptomatic. Diagnosis usually occurs by chance after routine hematologic evaluation, during antenatal screening or part of a family study. α-thalassemia trait results when 2 genes are deleted; patients usually have mild anemia with microcytosis but remain clinically asymptomatic. Hemoglobin H (HbH) disease can be caused by a 3 gene mutation or 2 gene mutation in combination with another globin mutation (such as Constant spring) resulting in less than 30% α-globin gene expression. Patients experience hemolytic anemia with varying degree with of hypersplenism, jaundice and other complications such as gallstones or infections. Patients with a milder HbH phenotype may be managed by intermittent blood transfusions during periods of stress or intercurrent illness. More severe cases characterized by severe hemolytic anemia require regular blood transfusions and chelation therapy. When no α-globin is produced, infants suffer from Hb Bart’s Hydrops Fetalis Syndrome characterized by severe intra-uterine anemia, marked hepatosplenomegaly, cardiac failure, ascites and growth retardation. Affected neonates, most often the proband, usually die in utero or shortly after birth (Harteveld and Higgs 2010).

### Pathophysiology of sickle cell disease

Sickle cell disease is an autosomal recessive disorder caused by a point mutation in the β-globin chain resulting in the single amino acid substitution of valine rather than glutamic acid at position 6 (Ingram 1956). The inheritance of β^S^ from both parents results in the most common and severe form of the disease, Hb SS. However, other compound heterozygous forms of the disease including HbSC, HbS/β^0^thalassemia and HbS/β^+^-thalassemia result in sufficient expression of HbS to cause intracellular sickling (Ware et al. 2017). The abnormal S hemoglobin causes aberrant β-chain formation so that during episodes of deoxygenation, HbS molecules polymerize together to force the normal biconcave–shaped erythrocyte into an elongated, rigid form (Acquaye et al. 1988). Sickled erythrocytes cause vaso-occlusion in capillaries and arterioles as well as abnormal endothelial interactions and chronic hemolysis^38^. This results in anemia and tissue hypoxia which results in a variety of acute complications including painful vaso-occlusive crises (VOCs), stroke, priapism, and acute chest syndrome (ACS). Chronic complications are related to small and large vessel vasculopathy, progressive ischemic organ damage and chronic hemolysis; these include cerebrovascular disease, retinopathy, pulmonary hypertension, gallstones renal failure, hyposplenism, bone disease, hepatopathy and premature death (Piel et al. 2017; Powars et al. 2005).

### Polycythemia vera

Polycythemia vera (PV) is a clonal disorder of myeloproliferation in the bone marrow. It is characterized by increased red cell mass associated with the proliferation of the erythroid, megakaryocytic and granulocytic cell lines. It is the most common chronic myeloproliferative neoplasm (MPN) in adults and is virtually always characterized by the JAK2V617 point mutation corresponding to JAK2 exon 14 and less commonly, exon 12. The mutation results in the constitutive activation of the JAK/STAT signaling pathway that is independent of EPO signaling (James et al. 2005; Baxter et al. 2005; Kralovics et al. 2005). There are a multitude of downstream effects including increased phosphorylation of STAT5 in erythroid progenitors and activation of PI3K and MAPK/ERK pathways. Furthermore, JAK2 can enter the nucleus and phosphorylate histone H3 which exerts a direct effect on the regulation of gene expression.

Early disease manifestations are primarily related to complications from hyperviscosity including peripheral arterial and venous thrombosis, stroke and myocardial infarction. While the primary cause of mortality is attributed to thrombotic complications, progression to myelofibrosis and acute leukemias remain a significant cause of long-term morbidity and mortality (Passamonti et al. 2003; Geyer et al. 2016; Tefferi et al. 2013).

### Anemia of chronic disease

Elevated levels of circulating inflammatory mediators such as interleukin 6 (IL-6), transforming growth factor-β (TGF-β), tumor necrosis factor (TNF) and interferon-γ (IFN-γ) are found in a multitude of chronic inflammatory conditions and malignancies (Landskron et al. 2014; Boutou et al. 2012). These conditions are often characterized by anemia which suggests a mechanism for cytokine-mediated inhibition of erythropoiesis (Freireich et al. 1957; Johnson et al. 1989; Means and Krantz 1993; Libregts et al. 2011)**.** Anemia of chronic disorders (ACD) is the 2nd most common form of anemia worldwide and is associated with significant impairments in quality of life (Locatelli et al. 2004). The pathophysiology is multifactorial and involves several different pathways.

IL-6 significantly alters iron trafficking via induction of hepcidin expression which results in the blockade of cellular iron egress and reduced iron availability for erythropoiesis (Weinstein et al. 2002; Nemeth et al. 2004a). Iron homeostasis is further disrupted as several pro-inflammatory cytokines including interleukin 1 (IL-1), IL-6, interleukin 10 (IL-10) and TNF stimulate the uptake of iron by macrophages via various mechanisms including the stimulation of erythrophagocytosis. Alongside this, IFN-γ and lipopolysaccharides have been shown to suppress the expression of ferroportin mRNA thereby leading to the retention of iron within monocytes (Ludwiczek et al. 2003; Fahmy and Young 1993). Together, these events all lead to the blunting of dietary iron absorption and increased iron retention in macrophages which is reflected by hypoferremia and normal or increased ferritin levels. In addition, inflammation negatively affects the synthesis and biological activity of EPO. This phenomenon is thought to be due to the combination of direct inhibition via cytokines such as TNF and IL-1 (Leng et al. 1996) as well as reduced EPO-R expression on erythroid progenitors (Wang et al. 1995; Taniguchi et al. 1997).

Finally, pro-inflammatory cytokines, especially the interferons and TNF appear to inhibit the proliferation and differentiation of erythroid progenitor cells leading to ineffective erythropoiesis (Papadaki et al. 2002; Pontikoglou et al. 2006). More recently, elevated levels of serum high-mobility group box 1 (HMGB1) was found in a murine model of sepsis and administration of anti-HMGB1 monoclonal antibodies significantly ameliorated the development of anemia. Furthermore, administration of recombinant HMGB1 to healthy mice mediated the anemia and extramedullary erythropoiesis with a significant elevation in reticulocyte counts. This suggests the role of HMGB1 as a mediator of anemia of chronic disease and suggests a potential therapeutic strategy for anemia in sepsis (Valdés-Ferrer et al. 2015) and chronic disease.

## Treatment strategies

The diseases described above are not meant to be a comprehensive compendium of red cell disorders but rather examples of defective erythropoiesis that, in their treatment, demonstrate the application of newer therapeutic strategies (Tables 1, 2 and 3). Although not always successful in clinical trials, these new approaches clearly define the path forward.

**Table 1:** Novel therapeutics in Sickle Cell Disease

a. Gene therapy						
Bluebird Bio	BB305 lentiviral vector (betibeglogene darolentivec)	Anti-sickling β-globin	- Severe SCD - age ≥ 18 years	IV	NCT02140554	Open - Phase 1/2
Children’s Hospital Medical Center, Cincinnati	Gamma globin lentiviral vector	Anti-sickling γ-globin	- Severe SCD - age 18–35	IV	NCT02186418	Open - Phase 1/2
b. Small molecule targets						
Boston University	SIRT1	HbF induction				Early stage of development - pre-clinical
The Cleveland Clinic	Decitabine and tetrahydrouridine	HbF induction	- age ≥ 18 years - HbSS, HbSβ°, HbSβ^+^, HbSC	Oral	NCT01685515	Completed - Phase 1
Celgene	Pomalidomide	HbF induction	- age 18–60 - HbSS, HbSβ°	Oral	NCT01522547	Completed - Phase 1
Novartis Pharmaceuticals	Panobinostat	HbF induction	- age ≥ 18 yers - HbSS, HbSβ°	Oral	NCT01245179	Open - Phase 1
Dana Farber Cancer Institute	Vorinostat (Zolinza)	HbF induction	- age 18–60 - HbSS, HbSβ°	Oral	NCT01000155	Discontinued - Phase 1/2

**Table 2:** Novel Therapeutics in β-Thalassemia

Company	Drug/Target	Mechanism	Eligibility	Route	Clintrials.gov	Status
a. Gene therapy						
Bluebird Bio	BB305 lentiviral vector (betibeglogene darolentivec)	Improved erythropoiesis	- Transfusion-dependent β-Thalassemia -age 12–35	IV	NCT01745120	Active, not recruiting - Phase 1/2
IRCCS San Raffaele	GLOBE lentiviral vector	Improved erythropoiesis	- Transfusion-dependent β-Thalassemia -age ≥ 3 and < 65	IV	NCT02453477	Open - Phase 1/2
b. Small molecule targets						
New England Research Institutes	Decitabine	HbF induction	- age ≥ 18 - TD β Thalassemia and HbEβ-Thalassemia	Subcutaneously	NCT00661726	Completed - Phase 2
Medical College Kolkata	Decitabine	HbF induction	- age ≥ 18 - TDT and NDTD HbEβ-Thalassemia	Subcutaneously	–	Completed - ASH 2017
Novartis Pharmaceuticals	INC424 (Ruxolitinib)	Jak 1/2 inhibitor	- age ≥ 18 - TDT β- Thalassemia - Splenomegaly - iron chelation × 4 weeks	Oral	NCT02049450	Completed - Phase 2a
Acceleron	ACE-536 (Luspatercept)	Ligand trap TBG beta superfamily	- age ≥ 18 - TD and NTDT β-Thalassemia	Subcutaneously	NCT02268409	Active, not recruiting - Phase 2
Celgene	ACE-011 (Sotatercept)	Ligand trap TBG beta superfamily	- age ≥ 18 - TD and NTDT β-Thalassemia	Subcutaneously	NCT01571635	Active, not recruiting - Phase 2a

*NTDT* non-transfusion-dependent thalassemia

*TDT* transfusion-dependent thalassemia

**Table 3:** Novel therapeutics in Polycythemia Vera

Company	Drug	Mechanism	Eligibility	Route	Clintrials.gov	Status
Small molecules targets						
Incyte Coproration Novartis	Jak 1/2 inhibitor (ruxolitinib) vs BAT	Cytoreduction	- age ≥ 18 years	Oral	NCT01243944	Active, not recruiting - Phase 3
Incyte	Jak 1/2 inhibitor (ruxolitinib) vs HU	Cytoreduction	- age ≥ 18 years	Oral	NCT01632904	Completed - Phase 3
AOP Orphan Pharmaceuticals AG	Pegylated interferon alpha-2b (AOP2014) vs HU	Cytoreduction	- age ≥ 18 years	subcutaneously	NCT01949805	Completed - Phase 3
Roskilde University Hospital,	Vorinostat	Cytoreduction	- age ≥ 18 years	Oral	–	Completed - Phase 2
Italframaco	Givinostat vs HU	Cytoreduction	- age ≥ 18 years	Oral	NCT00928707	Completed - Phase 2

*BAT* best available therapy

HU hydroxyurea

Hemoglobinopathies are the most common monogeneic disorders worldwide, with approximately 7% of the population identified as genetic carriers (Kohne 2011). Sickle cell disease and β-thalassemia are two of the most common genetic disorders affecting red blood cell (RBC) development (Weatherall et al. 2006). The hallmarks of these two diseases involve absent, or aberrant β-globin chain formation resulting in ineffective erythropoiesis. At present, allogeneic hematopoietic stem cell transplantation (HSCT) is the only established definitive curative option for SCD and β-thalassemia. Overall survival for both diseases following HSCT now approaches 90%. Unfortunately, the majority of patients do not have matched sibling donors available necessitating the use of matched-unrelated donor (MUD) transplants. Historically, MUD bone marrow transplantation has been associated with significant morbidity and mortality resulting from graft-versus-host disease (GvHD) and graft failure (Angelucci et al. 2014; Bacigalupo 2012).

Drugs that target the induction of fetal hemoglobin have been the prototypical strategy used to manage the sequelae of sickle cell disease including vaso-occlusion and anemia. This is based on early observational studies that demonstrate decreased mortality in patients with higher levels of Hb F (Leikin et al. 1989; Platt et al. 1994). Currently, Hydroxyurea is the most widely used disease-modifying therapy for sickle cell disease in children (Brawley et al. 2008). Given that observational studies of patients with β-thalassemia and pancellular hereditary persistence of fetal hemoglobin (HPFH) have milder disease phenotype (Musallam et al. 2012), alternative strategies to more effectively enhance HbF production are attractive therapeutic targets for both SCD and β-thalassemia.

Several other investigational drugs have been shown to increase fetal hemoglobin and are in various stages of clinical investigation. Alternatively, gene therapy is an attractive therapeutic modality that represents a paradigm shift in the treatment of hemoglobinopathies away from conventional medication and symptom alleviation toward a curative approach. Here we describe the current state of therapy for these diseases as well as areas of active investigation.

### Gene therapy

The following prerequisites are required for successful gene therapy in β-hemoglobinopathies:

1. Efficient gene transfer with high HSC engraftment,
2. Consistent gene expression independent of the site of integration,
3. High expression of globin gene expression (β or γ),
4. Erythroid lineage and developmental stage- specific expression of transferred globin gene, safe integration and expression of gene with little to no risk of insertional oncogenesis (Chandrakasan and Malik 2014).

Gene therapy exploits the ability of retroviruses (RV) to reverse transcribe their RNA into complementary DNA (cDNA) which can then be incorporated into the host cell genome for therapeutic delivery of gene elements. Most successful gene therapy trials utilize lentivirus (LV) vectors as they possess the ability to enter into an intact nucleus and integrate into non-dividing cells. This results in high efficiency transduction of genetic material. Furthermore, LVs are self-inactivating such that all viral transcriptional machinery is removed once genetic material is transfected into the host cell.

Hemoglobinopathies require sufficiently high levels of globin genes expression for therapeutic correction, thus posing an additional challenge for successful gene therapy. Identification of critical regulatory elements required for high β-globin gene expression has resulted in feasible gene therapy options.

LV vectors carrying a modified β globin gene with anti-sickling properties have been shown to be effective in both SCD and β-thalassemia murine models (Persons et al. 2001; Pawliuk et al. 2001). SCD patients with increased levels of HbF have long been shown to have a milder disease phenotype (Powars et al. 1989). Furthermore, fetal hemoglobin is a more potent anti-sickling hemoglobin as compared to adult hemoglobin (Sunshine et al. 1978), thus forming the basis for developing vectors containing γ -globin gene cassettes. Several LV-based vectors have been developed that utilize γ -globin cassettes (Persons et al. 2003; Pestina et al. 2009) with one model utilizing γ -globin coding sequences with β-globin regulatory elements (Perumbeti et al. 2009).

#### Gene therapy in Thalassemia

The first successful correction of thalassemia with an LV vector was reported in a murine model of β -thalassemia intermedia. There was an average increase in hemoglobin by 3–4 g/dL per LV vector copy with correction of red cell indices (May et al. 2000). The first successful human gene therapy trial was conducted in June 2007 for a transfusion-dependent patient with Hemoglobin E/β-thalassemia (HbEβ0). The patient received a myeloablative conditioning regimen with busulfan followed by infusion with a SIN LV-based β globin vector (βT87Q) – transduced into CD34+ cells. The patient achieved transfusion-independence with a stable hemoglobin of 8.5–9.0 g/dL by 2 years post infusion. Insertion site analysis initially demonstrated clonal expansion of erythroid cells (10–12%) at the high mobility group AT-hook 2 (HMGA2) locus. While this clone peaked at 4% of hematopoietic cells, it has since declined to approximately 1% at 5 years post transplantation without an associated reduction in total hemoglobin. The leukemogenic potential of this clone is yet to be elucidated (Cavazzana-Calvo et al. 2010). With enhanced LV vector transduction efficiency, further data from this group have demonstrated much higher transgene expression resulting in an increase in hemoglobin by 4–6 g/dL within 2–5 months following transplantation. At up to 3 years follow up, no clonal events have been described for these patients (Kwiatkowski et al. 2017).

#### Gene therapy in SCD

The first SCD patient treated with gene therapy was conducted in a 13 year-old male on a chronic transfusion program for recurrent VOCs, silent infarct, and acute chest syndrome (ACS). Similarly, he received myeloablative conditioning with busulfan followed by infusion with the βT87Q LV-based B-globin vector. The subject did not experience any vector-related side effects nor any SCD-related hospitalizations despite discontinuing his chronic transfusion program. 30 months post transplantation he was hospitalized for management of VOC in the setting of an acute viral illness. His most recent total hemoglobin is 12.4 g/dL with no evidence of clonal dominance (Ribeil et al. 2017).

An additional nine patients with severe SCD have since received the Lentiglobin drug product with a fully myeloablative conditioning regimen. To date, there have been no severe adverse effects attributable to the drug product (Kanter et al. 2017). While longer term data is not yet available for these subjects, two patients have discontinued their chronic transfusion regimen with modest improvements in hemoglobin and stable expression of the vector 6 months post infusion (Cavazzana et al. 2017).

Multiple clinical trials for gene therapy of β -thalassemia (Mansilla-Soto et al. 2016; Marktel et al. 2017) or severe SCD (Archer et al. 2015) with lentiviral vectors have since emerged from several centers. The majority use a recombinant β-globin gene, with anti-sickling properties for SCD, combined with a myeloablative conditioning regimen. There is currently one open trial utilizing a γ -globin vector for severe SCD); interim results are not yet available.

The predominant trend, overall, for these trials include transfusion independence in β-thalassemia and amelioration of disease phenotype in severe SCD. Transplantation is generally well tolerated with side effects attributed to the conditioning regimen and no major (≥ grade 3) toxicities attributed to the LV vector.

### Gene editing

Given ongoing safety concerns regarding oncogenesis and clonal expansion following vector insertion into the human genome, gene editing technologies remain an attractive therapeutic modality. Genome-editing therapies exploit the ability of the human genome to repair itself following double-strand breaks (DSBs). DSBs are repaired via homology-direct repair (HDR) pathways or non-homologous end joining (NHEJ). The HDR pathway is utilized to insert custom sequences into the genome via an engineered endonuclease co-delivered with an extrachromosomal repair template. These approaches employ a transient ex vivo intervention and do not result in permanent insertion of foreign DNA into the genome. Currently, Zinc-finger nucleases (ZFN), transcription activator-like effector nucleases (TALENs) and clustered regulatory interspersed short palindromic repeat (CRISPR/Cas) endouncleases are the systems available to make site-specific DSBs. Early proof-of-concept studies have been reported for ZFN and TALEN-mediated correction of α-thalassemia (Chang and Bouhassira 2012), β-thalassemia (Ma et al. 2013) and SCD (Sebastiano et al. 2011). Similarly, high fidelity ß-globin gene editing has been described utilizing CRISPR/Cas9 –based targeting in SCD (Hoban et al. 2016; Dever et al. 2016).

With continued technique optimization, improved editing efficiencies and cell viability have been achieved to merit expansion into clinical trials. Lin et al. (2017) describe their success with CRISPR/Cas9-based editing of human primary hematopoietic stem and progenitor cells (HSPCs). This technology was employed to re-created specific genetic variations associated with hereditary persistence of fetal hemoglobin to induce HbF expression. HSPCs from healthy donors and patients with SCD and β-Thalassemia demonstrated clinically relevant increases in γ-globin mRNA that have persisted at 16 weeks. Many additional pre-clinical studies utilizing primarily CRISPR/Cas9-based gene editing strategies have emerged, which hold promise for translation into clinical trials (deDreuzy et al. 2017; Lux et al. 2017; Dever et al. 2017; Yu et al. 2017)

Inducible pluripotent stem cells (iPSC) utilize reprogramming of genes to induce the multilineage differentiation potential of mature somatic cells. This system is advantageous as it allows for screening of the ideal clone with safe integration and high gene expression profile. In vitro experiments with human iPSCs (hiPSCs) have been hampered by their inability to demonstrate terminal erythroid differentiation with mature, enucleated, β-globin- expressing erythroid cells. Utilizing optimized cell processing techniques, Rosanwo et al. (2017) have generated conditionally immortalized hematopoietic progenitors from SCD patients, capable of robust terminal differentiation. When transplanted into immunodeficient mice, these lines underwent globin switching with a 27% induction of β- globin expression. These results hold promise for future disease modeling and development of novel therapeutic treatments for all hemoglobinopathies.

This technology has been further developed via the combination of hiPSC-based cell replacement therapies with gene editing techniques for monogeneic disease correction. This approach involves generating iPSCs from patients with a disease of interest and utilizing the above mentioned gene editing strategies i.e. TALEN, CRISPR/Cas, ex-vivo to repair a mutated gene or induce expression of protein necessary to remediate disease burden (Hockemeyer and Jaenisch 2016). Several studies have been piloted for correction of SCD (Huang et al. 2015; Sun and Zhao 2014) and β –Thalassemia (Xu et al. 2015).

### Small molecule targets

#### Jak inhibitors

In considering the importance of the JAK/STAT pathway in erythropoiesis and in particular, phosphorylation of STAT 5 during stress erythropoiesis, JAK inhibitors are an obvious therapeutic target in disorders of erythropoiesis (Porpiglia 2012). Ruxolitinib is a selective JAK 1/2 inhibitor that was first approved in 2011 for use in myelofibrosis. It was the first JAK inhibitor FDA approved for clinical use and has since become the cornerstone of treatment for intermediate or high-risk myelofibrosis (Verstovsek et al. 2017).

##### Polycythemia Vera

The discovery of the JAK2^V617F^ mutation in 2005 has led to a deeper understanding of the molecular mechanism underpinning PV and has since allowed for the development of targeted therapy options. The safety and efficacy of ruxolitinib in patients with PV with splenomegaly who are resistant to or intolerant of hydroxyurea were explored in a phase III (RESPONSE) clinical trial. Results demonstrated superiority as compared to best available therapy in controlling hematocrit, reducing spleen volume and improvement in symptoms >including pruritis and night sweats. JAK2^V617F^ allele burden decreased from baseline and declined steadily over time (maximal mean change, − 34.7% at week 112). Grade ≥ 3 toxicities were limited to anemia and thrombocytopenia in 2 and 3% of patients, respectively and 6% of patients developed herpes zoster infections (0% in control arm) (Vannucchi et al. 2015). In the phase IIIb RELIEF trial, the benefit of switching to ruxolitinib in patients with stable hematocrit on hydroxyurea but with persistent PV-related symptoms i.e. fatigue, pruritis and muscles aches failed to demonstrate any significant benefit (Mesa et al. 2017). The over-arching relationship between JAK2^V617F^ allele burden and disease progression remains unclear, and warrants further population-based cohort studies to assess this.

Phlebotomy and hydroxyurea remain the cornerstone of treatment with the aim to prevent cardiovascular complications via reduction of hematocrit and associated blood viscosity. Ruxolitinib, is effective in patients who are intolerant to phlebotomy/hydroxyurea or become resistant and is currently approved as second line therapy. Interferon-α has been shown to induce molecular remission via unclear mechanisms. A phase III clinical trial comparing pegylated interferon α to hydroxyurea was completed in July 2016. The trial demonstrated non-inferiority for pegylated interferon α with a superiority safety profile and increased rate of complete remission following end of therapy (Gisslinger et al. 2016).

##### β -*Thalassemia*

In their mouse model of thalassemia major and thalassemia minor, Libani et al. (2008) demonstrated a disproportionate percentage of erythroid cells remaining in S phase with an immature erythroblast morphology. When treated with an oral Jak2 inhibitor, thalassemic mice experienced marked reduction in spleen size and a decreased ratio of immature to mature erythroblasts. Several other murine studies have demonstrated a significant remediation of their splenomegaly, with a trend towards normalization of the splenic architecture and a decrease in the proportion of splenic erythroid progenitors (Casu et al. 2011; Casu et al. 2016a). A multicenter, single arm phase II clinical trial (TRUTH study) using ruxolitinib for regularly transfused thalassemia patients demonstrated a trend towards reduction in requirement for transfused RBCs and a slight increase in the pre-transfusion hemoglobin. There was a reduction in mean spleen volume from baseline (− 26.8% by week 30). The drug was well tolerated with no significant safety issues, however phase III clinical trials have not yet commenced (Aydinok et al. 2016).

#### Activin Signalling

Activins are soluble ligands, expressed in various tissues that belong to a large group of proteins called the transforming growth factor β (TGF-β) family. Activin expression, particularly activin A and bone morphogenic protein (BMP) 2 and 4 have been shown to have a role in the regulation of erythropoiesis (Maguer-Satta et al. 2003). Luspatercept (ACE-536) and Sotatercept (ACE-011) are recombinant receptor antagonists that block the binding of TFG-β family ligands to the ActrIIB and ActrIIA receptors, respectively.

##### β-*Thalassemia*

Early mouse studies utilizing murine orthologs of these drugs demonstrated improved anemia and increased maturation of erythroblasts consistent with the amelioration of ineffective erythropoiesis (Langdon et al. 2015; Carrancio et al. 2014; Suragani et al. 2014a; Suragani et al. 2014b). These effects have been replicated for both luspatercept and sotatercept in phase II clinical trials for patients with β-thalassemia, demonstrating decreased transfusion requirements in transfusion- dependent patients, an increase in hemoglobin among non-transfusion dependent patients and a reduction in iron overload (as evidenced by a decrease in liver iron concentration (LIC)). Both drugs were generally well tolerated with no serious adverse effects reported. The most common adverse effects were mild to moderate musculoskeletal pain and headaches (No Author (2018); Piga et al. 2016; No author (2018)). A phase III, multi-center double-blind trial (BELIEVE) is ongoing to evaluate the efficacy and safety of luspatercept (ACE-536) in transfusion- dependent thalassemia patients (No author (2018)).

#### Histone deacetylase inhibitors

Histone deacetylases (HDACs) are part of a diverse group of enzymes that regulate gene expression through chromatin modification. Histone acetylation relaxes chromatin structure, thereby increasing the accessibility of transcription factors to their target genes and increases gene expression (Haberland et al. 2009).

##### Polycythemia Vera

High levels of histone deacetylase activity have been demonstrated in patients with PV (Skov et al. 2012). Vorinostat is a pan-HDAC inhibitor that has been shown in a phase II clinical trial to decrease the JAK2 V617F allele burden, reduce splenomegaly and normalize the leukocyte and platelet counts. 52% of patients discontinued the study drug before the end of the study period due to unacceptable toxicity with the most common side effects being diarrhea, fatigue renal impairment, nausea and hair loss (Andersen et al. 2014).

Givinostat is an oral HDAC class I and II inhibitor that is currently under investigation for efficacy and safety in PV patients. Early phase II trials in patients unresponsive to the maximal tolerated dose of hydroxyurea (HU) demonstrated a significant hematologic response and resolution of grade ≥2 pruritus and splenomegaly. The combination of givinostat and HU was generally well tolerated, however premature study termination occurred in 18% of patients and grade ≥ 3 toxicity occurred in two patients (Finazzi et al. 2013).

##### Sickle cell disease

Preclinical studies have demonstrated elevated Hb F with nonspecific HDAC inhibition (Shearstone et al. 2016; Esrick et al. 2015). The safety and tolerability of vorinostat was recently demonstrated in a phase 1 study of 5 sickle cell patients. Although only 1 patient met criteria for success (4% absolute increase or a 100% relative increase in HbF %), the drug was well tolerated and a phase II trial is warranted to establish an optimal dosing profile (Okam et al. 2015). A phase I clinical trial is currently in process for panobinostat, a pan-HDAC inhibitor in adult patients with severe SCD. Preliminary results are not yet available. Lastly, at low, non-toxic concentrations, Givinostat was shown to induce HbA and HbF in erythroid cells from SCD patients at a level comparable to that of HU and butyrate. These results support the need for further evaluation of givinostat as a new candidate molecule for the treatment of hemoglobinopathies.

Other HDAC inhibitors including derivatives of butyrate have shown efficacy in clinical studies but are limited by the need for parenteral administration (Atweh et al. 1999; Perrine et al. 1993; Pace et al. 2002). 2,2-dimethylbutyrate (HQK-1001) is the first oral butyrate derivative that that initially showed promise in pre-clinical trials. Unfortunately, the phase II clinical trial (NCT01601340) was terminated after interim analysis did not show any significant increase in fetal hemoglobin and worsening VOC in the experimental group (Reid et al. 2014).

#### Hypomethylating agents

DNA-methyl transferases are a family of enzymes that catalyze the transfer of methyl groups to cytidine nucleotides of genomic DNA. DNA-methyl transferase 1 (DNMT1) is a chromatin-modifying enzyme that maintains methylation marks on DNA throughout cell division. Methylation of DNA is important for epigenetic gene regulation and has been shown to silence genes that direct the epigenetic silencing of the HbF gene, specifically BCL11A (Zhou et al. 2010). 5- azacitidine and 5-aza-2′-deoxycytidine (decitabine) are cytidine analogues that cause DNA hypomethylation via inhibition of DNMT1 and can lead to an increase in HbF. Decitabine is FDA approved for the treatment of myeloid malignancies, however it can cause cytotoxicity at high doses.

##### Sickle cell disease

In small scale animal (DeSimone et al. 1982) and patient studies (Ley et al. 1983; Charache et al. 1983), decitabine and 5’azacitidine have been shown to successfully increase fetal hemoglobin production in patients with SCD. The first human clinical trial aimed to pharmacologically re-induce HbF via DNMT1 inhibition began in September 2012. The phase I trial combined decitabine (to deplete DNMT1) with tetrahydrouridine (to increase decitabine half-life and subsequent oral-bioavailability). This oral combination was well tolerated without any significant (≥ grade 3) toxicities encountered. Furthermore, decitabine produced significant increases in fetal hemoglobin with an increased proportion of HbF-enriched RBCs (F-cells) (Molokie et al. 2017). Further clinical trials are warranted to establish optimal dosing.

##### β*-Thalassemia*

The first trial examining the use of DNA hypomethylating agents in HbF induction was conducted by DeSimone et al. (DeSimone et al. 1982) utilizing 5-azactydine in phlebotomized baboons. A significant increase in HbF was noted within 5 days of therapy, which prompted Ley et al. (Ley et al. 1982) to test 5-azacytidine in a patient with severe β-Thalassemia. Within 7 days of treatment, γ -globin synthesis increased seven-fold and the patient demonstrated an increase in hemoglobin (8.0–10.8 g per deciliter). Due to ongoing safety concerns including myelosuppression, immunosuppression and cytotoxicity, subsequent use of 5-azacytidine has been limited to severe cases when conventional therapy is not feasible. Several small case reports have consistently reported rapid and favorable effects on HbF production and hematologic outcomes. Myelotoxicity requiring dose modification was the main adverse effect (Dunbar et al. 1989; Lowrey and Nienhuis 1993).

In their pilot study using decitabine for the treatment of hemoglobinopathies, Olivieri et al. (2011) demonstrated a modest improvement in total hemoglobin and HbF from baseline in all 5 patients. Furthermore, there was an overall decrease in the markers of ineffective erythropoiesis such as indirect bilirubin, LDH and reticulocyte count. The drug was well tolerated with the major side effect limited to an asymptomatic increase in platelet count.

The results of these studies are promising, however little progress has been made in developing larger scale clinical trials in order to truly study the benefits of HbF induction in hemoglobinopathies. Recently, Decitabine was trialed in both transfusion-dependent and transfusion-independent HbEβ-Thalassemia patients. The drug was efficacious in increasing HbF percentage in both groups and transfusion-dependent patients demonstrated an overall decrease in transfusion requirement. The drug was well tolerated with no documented hematologic toxicity (Kalantri et al. 2017)

#### Immunomodulatory drugs

Thalidomide is an FDA-approved immunomodulatory drug, originally developed for use in patients with multiple myeloma (MM). Lenalidomide (Len) and Pomalidomide (Pom) are newer generation analogs of thalidomide that have increased activity in multiple myeloma with an improved side effect profile. Len and Pom have been shown to reduce the transfusion burden in patients with MM and myelodysplastic syndrome (MDS) (Tefferi et al. 2009; Raza et al. 2008), making this an attractive class of drug for the potential treatment of the hemoglobinopathies.

##### Sickle cell disease

Len and Pom were shown to be potent inducers of HbF during erythroid differentiation in both a murine model of SCD (Meiler et al. 2011) and in in vitro human CD34+ cells from SCD donors (Moutouh-de Parseval et al. 2008). A phase 1 clinical trial examining the effects of Pom in severe SCD adult patients was completed in October 2012 and found elevated levels of HbF, F-cells and total Hb with no significant adverse effects (Swerdlow et al. 2013). Dulmovits et al. (2015) further demonstrated that Pom reverses γ -globin silencing via transcriptional reprogramming of early adult erythroid progenitors. The drug is promising, and could potentially be used for the induction of fetal hemoglobin which could be clinically beneficial to patients with SCD.

##### β-thalassemia

Based on the pre-clinical studies in SCD, several isolated case reports using thalidomide and its derivatives for patients with β-thalassemia major have recently emerged. All of these case reports demonstrate an improvement in hemoglobin and an increase in HbF; the drugs have been very well tolerated with an improvement in the overall clinical status of the patients (Aguilar-Lopez et al. 2008; Masera et al. 2010).

#### SIRT

SIRT 1 encodes a nicotinamide adenosine dinucleotide (NAD)- dependent deacetylase that functions to remove acetyl groups from many histone and non-histone proteins (Haigis and Guarente 2006). SIRT1 has been shown to have an important role in transcriptional regulation, including direct deacetylation of histones. SIRT1 can therefore promote alterations in the methylation of histones and DNA leading to repression of gene transcription. Dai et al. (Dai et al. 2017) sought to investigate whether SIRT1 plays a role in gamma globin gene repression. In their in vitro study, SIRT1 was shown to enhance gamma globin gene expression in cord blood human erythroblasts and reactivate silenced gamma globin genes in adult erythroblasts. Furthermore, SIRT1 was shown to β-globin gene cluster locus control region (LCR) looping to the HbF promoter, inhibiting the expression of known HbF suppressors, BCL11A, KLF1, HDAC1 and HDAC2. This early data suggests a role for SIRT1 in modulating gamma globin production via transcriptional reprogramming and its activators are potential therapeutic targets for induction of HbF. Clinical trials are warranted to assess the translational potential of this therapeutic modality.

### Targeting iron overload

Iron is critical for hemoglobin synthesis and plays a key role in the regulation of erythropoiesis. Patients with SCD and β-thalassemia frequently are treated with chronic transfusions resulting in significant iron overload and organ toxicity. Effective iron chelators exist, however require long-term use and efficacy is hindered by poor compliance The liver antimicrobial peptide hepcidin (HAMP) is the master regulator of systemic iron homeostasis. HAMP facilitates surface expression of ferroportin, an iron export protein that is largely expressed in enterocytes, hepatocytes and macrophages, and facilitates iron absorption (Nemeth et al. 2004b).

HAMP signaling is regulated by the BMP-Son of mothers against decapentaplegic (SMAD) signaling pathway which is further modulated by total body iron availability. Hemojuvelin (HJV) functions as a co-receptor and is required to fully activate the SMAD signaling pathway (Babitt et al. 2006). During inflammatory states, IL-6 and STAT 3 pathways have also been shown to modulate HAMP signaling (Wrighting and Andrews 2006). SMAD signaling is negatively regulated by transmembrane protease serine 6 (TMPRSS6), a serine protease that cleaves HJV, reducing the phosphorylation of SMAD and dampening HAMP expression (Babitt et al. 2006). Homozygous mutations in TMPRSS6 lead to iron-refractory iron deficiency anemia in humans (De Falco et al. 2010). HAMP is overexpressed in humans with β-thalassemia (Papanikolaou et al. 2005), making it an attractive therapeutic target to decrease iron overload and help ameliorate ineffective erythropoiesis.

Blockade of TMPRSS6 in β-thalassemia intermedia mice has demonstrated an increase in HAMP mRNA as well as significant decrease in biomarkers of iron overload (serum iron, transferrin concentration, LIC and spleen weight). It has also been suggested to ameliorate ineffective erythropoiesis as evidenced by improved anemia, decreased alpha chain aggregates and increased proportion of mature erythroid progenitors (Guo et al. 2013; Nai et al. 2012; Schmidt et al. 2013). Additional murine studies have examined the additive effects of combining TMPRSS6 blockade with conventional oral iron chelation. Results demonstrate improvement in anemia and a decrease in iron overload; however, the effects, while greater than oral chelation alone, are not additive as compared with the sole blockade of TMPRSS6 (Casu et al. 2016b; Schmidt et al. 2015). To date, TMPRSS6 blockade has not been trialed in human studies.

Erythroferrone (ERFE), is a newly described erythroid regulator that is produced by erythroid precursors and has been shown to be involved in the regulation of hepcidin expression. ERFE expression is greatly increased in a murine model of β-Thalassemia which contributes to hepcidin suppression and subsequent iron overload characteristic of this disease (Kautz et al. 2014). Additional studies have demonstrated that ablation of ERFE in ß-Thalassemia mice can fully restore hepcidin levels and normalize iron regulation (Kautz et al. 2015). The suppression of ERFE may be a future therapeutic target that could be used alone or in conjunction with conventional chelators to ameliorate anemia and combat iron overload.

## Conclusion

The past 20 years have seen tremendous advancements in knowledge of the regulation of steady state erythropoiesis and in that context advancement in the understanding of the myriad molecular mechanisms leading to disordered erythropoiesis. As a consequence, multiple, novel therapeutic modalities are currently in clinical trials, many but not all, with promising results. In particular many of these new drugs target erythroid signaling, inductive a suppressive as well as the epigenetic regulation of globin gene synthesis. Furthermore, advancements in lentiviral-mediated gene therapy offer an exciting paradigm shift in treatment options as well as a promise of cure. Further clinical trials are required to expand the scope of these new therapeutic developments for clinical use.

## Abbreviations

ACD: Anemia of chronic disorders
ACS: Acute chest syndrome
BFU-E: Burst forming unit- erythroid
BMP: Bone morphogenic protein
cDNA: Complementary DNA
CFU-E: Colony forming unit- erythroid
CRISPR/Cas: Clustered regulatory interspersed short palindromic repeat
DNMT1: DNA-methyl transferase 1
DSB: Double strand break
EPO: Erythropoietin
EPO-R: Erythropoietin receptor
ERFE: Erythroferrone
GvHD: Graft-versus-host disease
HAMP: Hepcidin antimicrobial peptide
HbEβ0: Hemoglobin E/β-thalassemia
HbH: Hemoglobin H
HDAC: Histone deacetylase
HDR: Homology-direct repair
hiPSC: Human inducible pluripotent stem cells
HJV: Hemojuvelin
HMGA2: High mobility group AT-hook 2
HMGB1: High-mobility group box 1
HPFH: Hereditary persistence of fetal hemoglobin
HSC: Hematopoietic stem cell
HSCT: Hematopoietic stem cell transplantation
HSPC: Hematopoietic stem and progenitor cells
HU: Hydroxyurea
IFN-γ: Interferon-γ
IL-1: Interleukin 1
IL-10: Interleukin 10
IL-6: Interleukin 6
iPSCs: Inducible pluripotent stem cells
LCR: Locus control region
Len: Lenalidomide
LIC: Liver iron concentration
LV: Lentivirus
MAPK: Mitogen-activated protein kinase
MDS: Myelodysplastic syndrome
MM: Multiple myeloma
MPN: Myeloproliferative neoplasm
MUD: Matched-unrelated donor
NAD: Nicotinamide adenosine dinucleotide
NHEJ: Non-homologous end joining
PI3K: Phosphoinositide 3-kinase
Pom: Pomalidomide
pRBC: Packed red blood cell
PV: Polycythemia vera
RBC: Red blood cell
RV: Retroviruses
SCD: Sickle cell disease
SMAD: Son of mothers against decapentaplegic
TALEN: Transcription activator-like effector nucleases
TGF-β: Transforming growth factor- β
TMPRSS6: Transmembrane protease serine 6
TNF: Tumor necrosis factor
VOC: Vaso-occlusive crises
ZFN: Zinc-finger nucleases
